# Curcumin Induces Apoptosis in EJ Bladder Cancer Cells via Modulating C-Myc and PI3K/Akt Signaling Pathway

**DOI:** 10.4021/wjon335w

**Published:** 2011-06-08

**Authors:** Jingyu Wang, Zhiping Wang, Hanzhang Wang, Junli Zhao, Zhewen Zhang

**Affiliations:** a Institute of Pathophysiology, School of Basic Medical Sciences, Lanzhou University, Lanzhou, 730000, China; b Institute of Urology, Second Hospital, Lanzhou University, Lanzhou, 730030, China; c School of Basic Medical Sciences, Lanzhou University, Lanzhou, 730000, China

**Keywords:** Curcumin, C-myc, PI3K/Akt signaling pathway, Bax, Caspase, Apoptosis, Bladder cancer

## Abstract

**Background:**

Cancer chemopreventive agent curcumin has been shown to possess cell growth inhibition and apoptosis induction properties in several types of cancer. However, the detailed molecular mechanisms of the compound remain far from clear in EJ bladder cancer cells.

**Methods:**

The effect of curcumin on EJ cell growth and apoptosis was detected by MTT assays and flow cytometry. The phosphorylation levels of PTEN, PDK1, Akt, GSK-3β, c-Raf, and Bad and the expression levels of c-myc, Bax, Bcl-2, caspase-9, caspase-7, caspase-3, and PARP following curcumin administration were examined by immunoblots.

**Results:**

Curcumin suppressed the growth of EJ cells in a time and concentration dependent manner. Immunoblot showed that curcumin increased expression levels of c-myc and inhibited the activation of PI3K/Akt pathway in a time-dependent manner in EJ cells. Activation of PTEN, GSK-3β, c-Raf, caspase-9, caspase-7, and caspase-3, cleavage of PARP, upregulation of Bad and Bax, and downregulation of Akt and Bcl-2 were also found in curcumin-treated EJ cells.

**Conclusions:**

These findings establish a mechanistic linkup or interaction between c-myc, Bax, Bad, Bcl-2, caspase cascades, PI3K/Akt pathway and curcumin- induced apoptosis of EJ cells, suggesting that c-myc and PI3K/Akt signaling pathway play important roles in curcumin-induced apoptosis of EJ bladder cancer cells.

## Introduction

Over the last several years the incidence of bladder cancer has been increasing [1]. Chemoprevention is regarded as one of the most potent and realistic anti-cancer approaches aimed at decreasing the morbidity and mortality of cancer by induction of various cancer cells apoptosis through regulation of Akt, c-myc, nuclear factor КB (NF-КB), cyclooxygenase-2 (COX-2), apoptotic and other pathways [2-10]. Carcinoma of bladder is an ideal model for examining and applying cancer chemoprevention strategies because the bladder is easily accessible and can be monitored without impairing tissue [11]. More natural and dietary compounds including curcumin have been recognized as cancer chemopreventive agents due to its non-toxic (doses up to 8 g/day for 3 months are still safe) and anti-carcinogenic properties [12].

Curcumin, a natural compound present in turmeric, a rhizome of the plant curcuma longa linn, is widely used as a coloring and flavoring agent in daily cooking preparation for centuries [13]. Growth inhibition and apoptosis induction are common mechanisms proposed for the antitumor effects of curcumin [14]. Resistance to apoptosis is a hallmark of cancer, thus triggering apoptosis is a promising approach for carcinoma prevention and therapy [15].

Apoptosis is a highly organized cell death process [16]. The oncogene c-myc plays a critical role in modulating cell survival and apoptosis, accordingly, avoiding the oncogenic potential of cells that acquire deregulated c-myc [17]. Akt is a serine/threonine kinase that promotes cell growth and blocks apoptosis. Phosphorylation of both Ser473 and Thr308 residues is essential for full activation of Akt [18]. The tumor suppressor, phosphatase and tensin homolog (PTEN) negatively regulate the activity of Akt and prevent the translocation and activation of phosphoinositide-dependent kinase 1 (PDK1) [19]. Activated Akt functions to promote cell survival by suppressing apoptosis via subsequent modulation of a wide range of target molecules, like Bax [20], Bad [21], caspase-9 [22], glycogen synthase kinase 3β (GSK-3β) [23], c-Raf [24] and Bcl-2 [25], which regulate apoptosis. There are some observations related to the inhibitory effects of natural products against Akt, such as indole-3-carbinol (I3C) [26] and genistein [27], et al. Because of Akt’s major role in the hindrance of apoptosis through multiple mechanisms and its aberrantly activation in many cancers, especially, in more than half of primary carcinoma of bladder [28], the inhibition of the PI3K/Akt signaling pathway has emerged as an effective means to induce apoptosis, implicating an attractive target of chemopreventive agents in bladder cancer prevention and treatment. The determination of activated caspase can also be regarded as a biochemical marker for apoptosis [29]. Caspases become active when cleaved. Relative-adaptor proteins promote the cleavage of initiator caspases (e.g., caspase-9), initiator caspases cleave effector caspases (e.g., caspase-7, -3), the effector caspases destruct all function to result in apoptotic events [30, 31]. Apoptosis is controlled by a complicated net of pro-apoptotic and anti-apoptotic effector molecules, such as Bax and Bcl-2. The lower ratio of Bcl-2/Bax drives the cleavage of caspases and facilitates the induction of apoptosis [32, 33].

Apoptosis is an intricate process, therefore, to date, the detailed molecular mechanisms of curcumin leading to the induction of apoptosis in human bladder cancer remain far from clear [34]. In this report, investigation of the expression status and relationship of these apoptosis-associated factors in EJ bladder cancer cells is of considerable importance. Our final results clearly revealed that the induction of apoptosis by curcumin is caspase-dependent and occurs via increasing the expression of c-myc and blockade of PI3K/Akt signaling cascades, altering the balance between pro-apoptotic and anti-apoptotic members of Bcl-2 family.

## Materials and Methods

### Tumor cell lines and culture conditions

EJ human bladder tumor cells were obtained from Institute of Urology, Second Hospital, Lanzhou University. Cells were cultured in DME medium supplemented with 10% heat-inactivated fetal calf serum and 1% Penicillin-streptomycin in 5% CO_2_ incubator at 37 °C. Curcumin was diluted in complete medium and the final concentration of DMSO was not more than 0.1% in the in vitro study.

### Materials

Curcumin (98% purity), and 3-(4,5-dimethyl-2-thiazolyl)-2,5-diphnyl-2H-tetrazolium bromide (MTT) were purchased from Sigma Chemical Co. (St. Louis, MO). Phospho-Akt pathway sampler Kit, Apoptosis sampler Kit and antibodies against c-myc, Bax, Bcl-2, phospho-Bad (Ser136) were purchased from Cell Signalling Technology, Inc. (Beverly, MA, USA).

### Cell viability assay

EJ cells were seeded at 8 × 10^4^ cells/ml in 96-well culture plates and then treated with different concentrations of curcumin. After 24h or 48h incubation, the cell viability was detected by MTT assay.

### Apoptosis detection

EJ cells (2 × 10^6^) were incubated with indicated concentrations of curcumin for 24h. The percentage of cells containing sub-G1 DNA content and apoptotic cell were analyzed by a Becton-Dickinson FACScan flow cytometer (San Jose, CA).

### Western blot analysis

EJ cells (2 × 10^6^) were cultured with indicated various doses of curcumin (0, 12.5, 25 and 50 µM) for indicated times. Cell extracts were lysed in RIPA buffer, protein concentrations in total cell lysate were measured using the Bradford assay with BSA as standard. Fifty mg of cell lysates were electrophoresed in 10% Tris–glycine polyacrylamide gels and then transferred onto polyvinylidene difluoride (PVDF) membranes. Protein bands were visualized on X-ray film using an enhanced chemiluminescence (ECL) Western blotting substrate (Pierce; Rockford, IL).

### Statistical analysis

Data are presented as mean ± SD. Data were analyzed using One-way analysis of variance (ANOVA) followed by Bonferroni’s method for multiple comparisons. A level of P < 0.05 was considered statistically significant.

## Results

### Curcumin reduced cell viability in EJ cells

**Figure 1:**
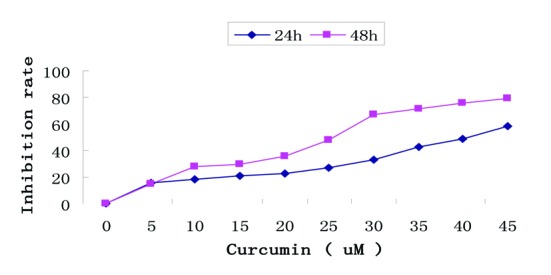
Growth inhibition by curcumin treatment in EJ cells. Dose- and time-dependent inhibitory effect of curcumin on EJ cells by MTT assay. Exponentially growing cells (8 × 10^4^ cells/ml) were seeded in 96-well plates and incubated with the working curcumin solutions at final concentrations of 5 - 45 µM for 24h and 48h. Data are represented as the means ± SD of at least three independent experiments performed in duplicate.

The anti-proliferative potential of curcumin in EJ human bladder cancer cells was assessed by MTT assay. As shown in Figure 1, curcumin induced a dose- and time-dependent reduction in cell viability compared with cells incubated in medium alone. Especially, cells exposed to the highest concentration (45 µM) exerted marked increase in cell growth suppression.

### Curcumin-induced apoptosis in EJ cells

**Figure 2:**
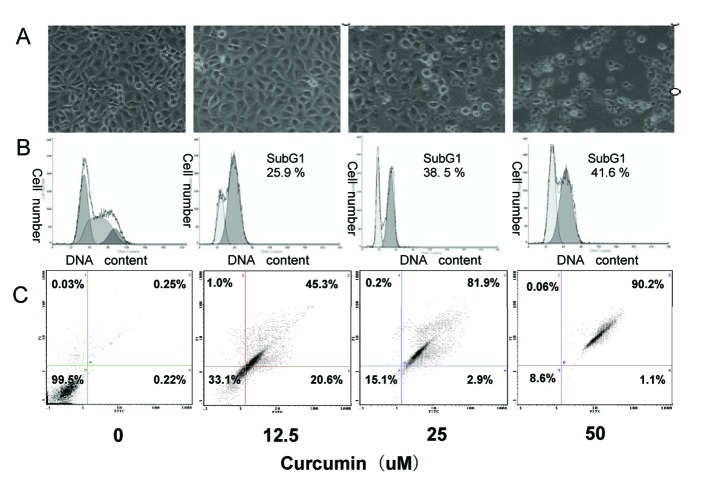
Induction of apoptosis in EJ cells by curcumin. EJ cells (2 × 10^6^ cells/ml) were treated with various concentrations of curcumin (0, 12.5, 25 and 50 µM) for 24h. (A) Morphological changes of EJ cells by treatment with curcumin. After 24h incubation, morphological changes of EJ cells were observed under the inverted phase-contrast microscope and photographed. The data are representative examples for duplicate tests. Original magnification, x 200. (B) Effect of curcumin on cell DNA content in cultured EJ cells. Treated-cells were washed, fixed, stained with PI and analyzed for DNA content by flow cytometry. The histograms presented are representative of at least three separate experiments. (C) Flow cytometric analysis of annexin V-FITC stained EJ cells after treatment with curcumin. Overall cells were stained with Annexin V-FITC and analyzed by flow cytometer for apoptotic events. At least three independent experiments were performed in duplicate.

In order to confirm whether the curcumin-induced growth inhibition was due to apoptosis in EJ cells, apoptosis-related experiments were performed. First, morphological change of EJ cells was observed with an inverted phase-contrast microscope after treatment with curcumin (0, 12.5, 25, 50 µM). The EJ cells showed remarkable morphological changes following increasing concentration of curcumin treatment. Cells treated with 25 µM curcumin grew less and were polarized and detached from the plate and floated with round-up shapes at higher doses (50 µM) (Fig. 2A). Furthermore, DNA content of curcumin-treated and untreated EJ cells was analyzed by flow cytometry. A prominent new peak, representing the sub-G1 peak, was raised following increasing concentration of curcumin treatment, which is indicator of apoptosis. The percentage of this sub-G1 peak in control and curcumin (12.5, 25, and 50 µM)-treated EJ cells was 0.43 ± 0.39%, 25.2 ± 1.1%, 37.5 ± 1.7% and 42.5 ± 0.76%, respectively (Fig. 2B), thereby suggesting apoptotic cell death. Then, we further used Annexin V-PI double staining experiment to identify the cell death types by flow cytometry. As shown in Figure 2C, EJ cells showed a dose-dependent apoptosis. Approximately 68 ± 1.3% of cells treated with 12.5 µM curcumin underwent apoptosis after 24h of exposure, as well as 85 ± 1.5% of cells treated with 25 µM curcumin and 91 ± 1.2% of cells treated with 50 µM curcumin. These results show, both quantitatively and qualitatively, that curcumin induces apoptosis in EJ cells.

### Curcumin induced apoptosis via upregulation of c-myc, thence modulating the activity of Bcl-2 and caspase family members

**Figure 3:**
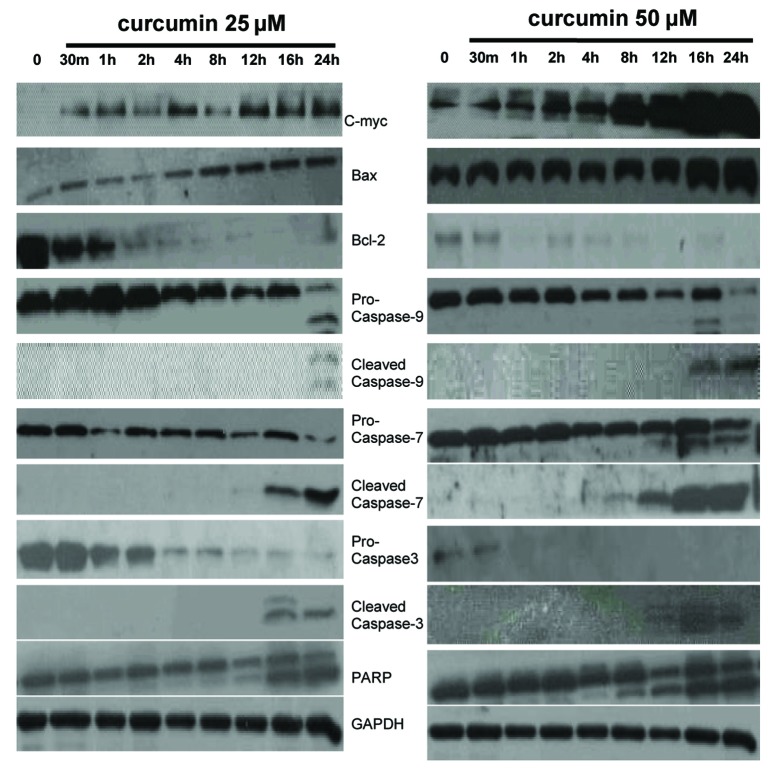
Expression of c-myc, Bcl-2 family members and intrinsic caspase cascade-related proteins in EJ cells. EJ cells (2 × 10^6^ cells/ml) were cultured in the absence or presence of curcumin (25 and 50 µM) for indicated times and whole-cell extracts were prepared. Then, 50 mg of protein extracts were isolated on 10% SDS-PAGE gel and subjected to western blot analysis with antibody against c-myc, Bax, Bcl-2, pro-caspase-9, cleaved caspase-9, pro-caspase-7, cleaved caspase-7, pro-caspase-3, cleaved caspase-3 and PARP. GAPDH was examined as a loading control. Data are representative of three independent experiments with similar results.

To make clear detailed mechanisms of curcumin-induced EJ cells apoptosis, we detected the expression of apoptosis-related proteins in curcumin (0, 25, and 50 µM)-treated EJ cells for indicated times. Firstly, as seen in Figure 3, we found that treatment with curcumin (25 and 50 µM) increased the expression of c-myc in a time-dependent manner as compared to vehicle treated EJ cells. There are reports in contrast to our findings in the literature that apoptosis could be induced through downregulation of c-myc in curcumin-treated Burkitt’s Lymphoma Raji cells [35], osteosarcoma cells [36], bladder cancer 253BJV cells [37], colorectal cancer cells [38], Hodgkin’s Lymphoma cells [39], HTLV-1-infected T-cell [40], and so on. The relevant records reported that the expression of Bcl-2, Bax and c-myc are concomitantly modulated. The cell death-promoting gene bax is directly downstream of c-myc in a pathway leading to apoptosis. Moreover, c-myc could be required for the activation of intrinsic caspase cascades [41-45]. Thus, further study is needed to elucidate the action of c-myc in curcumin-induced apoptosis. We investigated the expression of Bax, Bcl-2 and intrinsic caspase cascade-related molecules, like caspase-9, -7, -3. Our time-course experiment for immunoblotting assay of equal amounts of protein from medium treated and curcumin (25 and 50 µM)-treated EJ cells showed evident increase of Bax and decrease of Bcl-2 in time-dependent manner. Western blot data also showed that the levels of pro-caspase-9, -7, -3 proteins were decreased, but the levels of expression of the active cleavage of these caspases were increased in a time-dependent manner in curcumin-treated EJ cells as compared to vehicle treated cells. Subsequent immunoblotting analysis revealed that the cleavage of poly (ADP-ribose) polymerase (PARP) (the substrate of caspase-3, an early index of apoptosis) occurred in curcumin (25 and 50 µM)-treated EJ cells. Furthermore, the presenting time of the cleavage of caspase-9, -7, -3 and PARP following 50 µM of curcumin administration is earlier than that of 25 µM of curcumin treatment in EJ cells (Fig. 3), suggesting concentration dependent property of this effect. These data clearly indicated that c-myc might play a decisive role in the curcumin-induced apoptosis of EJ cells and that c-myc-induced apoptosis is intrinsic caspase-dependent.

### Curcumin induced apoptosis through inhibition of the PI3K/Akt signaling pathway in EJ cells

**Figure 4:**
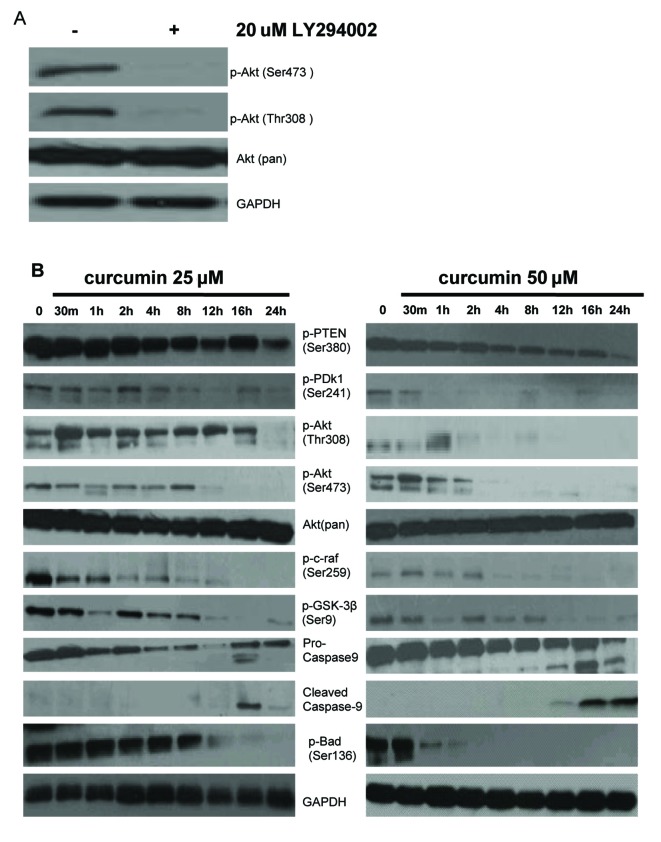
Effect of curcumin on PI-3K/Akt signaling pathway in EJ cells. (A) PI3K is required for constitutive Akt activation in EJ cells. EJ cells were treated with (+) or without (–) 20 µM LY294002 for 1h. Whole cell lysates were prepared and analyzed by western blot with antiphospho-Akt (Ser473), antiphospho-Akt (Thr308) and anti-Akt (Pan) antibodies. GAPDH was examined as a loading control. Data are representative of three independent experiments with similar results. (B) Effect of curcumin on the expressing of PI-3K/Akt signaling pathway proteins in EJ cells. EJ cells (2 × 10^6^ cells/ml) were treated with or without curcumin (25, 50 µM) for the indicated times and the cellular protein was analyzed by 10% SDS-PAGE gel and western blotting with antibodies to p-PTEN (Ser380), p-PDk1 (Ser241), p-Akt (Thr308), p-Akt (Ser473), Akt (pan), p-c-raf (Ser259), p-GSK-3β (Ser9), pro-caspase-9, cleaved caspase-9, and p-Bad (Ser136). GAPDH was examined as a loading control. Data are representative of three independent experiments with similar results.

We studied the involvement of the PI3K/Akt pathway in c-myc-induced EJ cells apoptosis. To test the status of Akt activation in EJ cells, phosphorylation of Akt was measured with corresponding specific antibody against phosphorylated Akt (Ser473 and Thr308) by Western blotting analysis. A high level of basal phosphorylated Akt (Ser473 and Thr308) in EJ cells was observed, phosphorylation of both sites (Ser473 and Thr308) is required for full activation of Akt. Total Akt levels were almost equal in EJ cells. Akt (Ser473 and Thr308) phosphorylation was dramatically weakened in EJ cells treated with the PI3K inhibitor LY294002 (20 µM) for 1h, which was PI3K-dependent. However, LY294002 had no effect on the total level of Akt (Fig. 4A). These results manifested that activated Akt plays a considerable role in cell growth and survival signaling of EJ cells. Consequently, we examined the related protein expression and phosphorylation level of PI3K/Akt signal transduction pathway after various concentrations of curcumin (0, 25 and 50 µM) treatment for indicated times in EJ cells. First, we detected the impacts of curcumin on phosphorylation status of proteins (PTEN and PDK1) that determine the activity of Akt. As shown in Figure 4B, curcumin reduced the expression of phosphorylated PTEN Ser380 (inactivated form of PTEN) and PDK1 (Ser241) in a time-dependent manner, indicating the activation of PTEN and inactivation of PDK1. Then, we tested the regulation of Akt phosphorylation using curcumin, the levels of Akt phosphorylation at Ser473 and Thr308 were significantly decreased in a time-dependent manner, but total Akt levels were unchanged following treatment with curcumin (25 and 50 µM) (Fig. 4B). Last, we also measured the effects of curcumin on phosphorylation status of proteins downstream of Akt. It is well known that Akt promotes cell proliferation and anti-apoptosis via modulating several crucial downstream signaling molecules including c-raf, GSK-3β, Bad, Bax and Caspase-9 [46]. The expression levels of c-raf (Ser259), GSK-3β (Ser9), Bad (Ser136) and pro-caspase-9 were decreased in response to curcumin (25 and 50 µM) administration in a time-dependent manner in EJ cells (Fig. 4B). The activity of GSK-3β, Bad and c-raf is inhibited by Akt through phosphorylations at Ser9, Ser136 and Ser259 respectively, which are deactivating modifications of these proteins. In other words, curcumin elevated the activity of GSK-3β, Bad, c-raf and caspase-9. Thus, the reduction of phosphorylated PDK1 and PTEN correlates with those of phosphorylated Akt, c-raf, GSK-3β, pro-caspase-9 and Bad. Furthermore, c-myc-induced apoptosis by curcumin was well related to the activation of caspase cascade (caspase-9) and Bcl-2 family proteins (Bad). These results reflected that aberrant PI3K/Akt signaling pathway could be directly and indirectly contributed to c-myc-induced EJ cell apoptosis.

## Discussion

There are data demonstrating that the bladder cancer is responsive to primary and secondary prevention efforts [11]. Chemopreventive agents including curcumin [7] operate their inhibiting effects on carcinogenesis by inducing apoptosis of tumor cells via affecting multiple signaling pathways including NF-КB, STAT3, MAPK and Bax induction [47-50]. However, to date, very little is known about the molecular mechanisms of curcumin-induced apoptosis in EJ bladder cancer cells. Our results showed that curcumin reduced the EJ cell viability in a time- and dose-dependent manner, which is related to induced apoptosis.

Accumulating evidence indicated that c-myc had an important function in cell proliferation and induction of apoptosis [51]. The previous report showed that apoptosis could be induced via downregulation of c-myc in curcumin-treated Burkitt’s Lymphoma Raji cells [35], osteosarcoma cells [36], bladder cancer 253BJV cells [37], colorectal cancer cells [38], Hodgkin’s Lymphoma cells [39], HTLV-1-infected T-cell [40], and so on. Interestingly, we obtained a steady increase in c-myc expression after 30 min treatment of curcumin (25 and 50 µM) in EJ bladder cancer cells in a time-dependent manner. The relevant documents reported that the expression of Bcl-2, Bax and c-myc is concomitantly modulated. Bax, a direct transcriptional target of c-myc, contributes to c-myc induced apoptosis, while the Bax-interacting prosurvival protein Bcl-2 inhibits this process. The loss of Bax expression in mice led to the loss of the apoptotic function of c-myc. C-myc could also be required for the activation of intrinsic caspase cascades [41-45]. We propose that in our study c-myc might play a decisive role in the EJ cell apoptosis induced by curcumin.

To determine the involvement of Bcl-2 family and caspase cascades in c-myc-induced apoptosis, the expression of related Bcl-2 family anti-apoptotic protein Bcl-2, pro-apoptotic members Bad and Bax, and intrinsic cascade pathway-associated molecules in curcumin-treated EJ cells were analyzed. Bcl-2 family proteins serve as key regulators in the pathway of apoptosis, which modulate the activation of intrinsic apoptotic pathway. Pro-apoptotic proteins (Bad, Bax) can trigger the intrinsic apoptotic cascades, while anti-apoptotic member (Bcl-2) prevents these changes [52]. Our results revealed that the administration of curcumin (25 and 50 µM) upregulated Bax, Bad protein and downregulated Bcl-2 molecules along with prolonged time. Simultaneously, the data demonstrated that curcumin increased the enzymatic activity of initiator caspase-9, subsequently activated the effector caspase such as caspase-7 and caspase-3, leading to the activation of intrinsic caspase cascades [53]. Similar to a later study in HL-60 cells [54], the induction of apoptosis by curcumin was also caspase-dependent in EJ cells. Among the caspase family members, caspase-3 is known to be one of the key executioners of apoptosis because caspase-3 activation causes the cleavage or degradation of downstream important substrates, like PARP, which is the hallmark of caspase-dependent apoptosis. Caspase-3 is essential for the morphological changes associated with apoptosis. Thus, the apoptosis induced by curcumin was also confirmed by the observation of cleavage of caspase-3 and PARP [55]. Our results showed that curcumin efficiently activated the expression of caspase-3, in turn cleaved PARP and induced apoptosis in EJ cells in a time-dependent manner. Nevertheless, further study is needed to explain the precise mechanism of c-myc-induced apoptosis in curcumin-treated EJ cells, we examined the expression levels of PI3K/Akt signaling pathway-related proteins. Because PI3K/Akt signaling cascade is related to cell survival via influencing the expression of Bcl-2 family members or caspase family proteins and other molecules, the limitation of full Akt activity could result in apoptosis [56], thus aberrant PI3K/Akt signaling pathway could be directly and indirectly contributed to c-myc-induced EJ cell apoptosis. The present data suggested that there was a high level of basal phosphorylated Akt (Ser473 and Thr308) in EJ cells. Blocking basal Akt activity by LY294002, a PI3K inhibitor, suggested that activated Akt plays a substantial role in growth and survival signaling of EJ cells. In the previous study, other investigators provided the evidence that 55% of primary tumors from patients with bladder cancer had markedly high level of phosphorylated Akt which is often associated with advanced forms of bladder cancer. These results support our observations. Thereby, Akt is a target of chemopreventive agents in bladder cancer prevention and treatment. Interestingly, we also found that curcumin and LY294002 can synergistically repress cell growth and induce apoptosis in EJ cells (data not shown). Currently, we are in the process of further study of this phenomenon trying to define the exact mechanisms.

Most widely reported as a tumor suppressor, PTEN may negatively regulate the PI3K/Akt pathway in cancer cells, which suggests that activated PTEN might decrease the expression of the PI3K/Akt pathway and promote apoptosis of cancer cells [57]. PDK1 is upstream of Akt, phosphorylated PDK1 may promote Akt which in turn modulates many downstream targets, including Bad, Bax, caspase-9, c-raf, GSK-3β and other proteins [46], which regulate cell survival and apoptosis. Dephosphorylation of Bad (Ser136) leads to targeting of Bad to mitochondria which hastens the apoptotic process. Bad can be phosphorylated at Ser136 by Akt, which inhibits the pro-apoptotic function of the protein [58]. GSK-3β is pro-apoptotic molecule with insults that activate the intrinsic mitochondrial apoptosis pathway. The PI3K/Akt signaling pathway is a major regulator of GSK-3β because Akt phosphorylates GSK-3β at Ser9 (inactive form of GSK-3β) [59], leading to apoptosis. Akt is known to affect the activity of Erk pathway by silencing c-Raf via phosphorylation at the inhibitory site of Ser259 [24], implicating crosstalk among different signaling pathways. Deactivation of Akt may increase the activity of caspase-9, leading to apoptosis through intrinsic caspase pathway. Our results indicated that phosphorylated PTEN at Ser380 (suppressive residue) could be attenuated by curcumin (25 and 50 µM), suggesting the activation of PTEN. Conversely, curcumin (25 and 50 µM) administration caused time-dependent inhibition of the levels of phosphorylated PDK1 and Akt. We also found that blocking Akt by curcumin remarkably decreased the levels of phospho-Bad, phospho-GSK-3β, phospho-c-Raf and pro-caspase-9 in EJ cells, indicating an increasing activity of Bad, GSK-3β, c-Raf and caspase-9. Although c-Raf was activated by curcumin in EJ cells, it is unknown whether sufficient activation of c-Raf activates the Raf/Erk cascades, because c-Raf activity is also regulated by protein-protein interaction [60], further research will be required. Accordingly, the suppression of anti-apoptotic PI3K/Akt signaling pathway by curcumin is also an important mechanism of action in c-myc-induced EJ cell apoptosis.

Taken together, these findings establish a complicated mechanistic linkup or interaction between c-myc, Bcl-2 family, caspase cascades, PI3K/Akt pathway and curcumin-induced EJ cell apoptosis, which may improve prevention outcomes for human bladder cancer. Although further in vivo investigation is underway in our laboratory, the present work suggests that curcumin may be a potential compound for the prevention and treatment of bladder cancer.
